# Predictive value of neutrophil-to-lymphocyte-ratio in neoadjuvant-treated patients with breast cancer

**DOI:** 10.1007/s00404-022-06726-7

**Published:** 2022-08-18

**Authors:** Alexandra von Au, Samra Shencoru, Lorenz Uhlmann, Luisa Mayer, Laura Michel, Markus Wallwiener, André Hennigs, Thomas Deutsch, Fabian Riedel, Joerg Heil, Michael Golatta, Andreas Schneeweiss, Florian Schütz, Christoph Domschke

**Affiliations:** 1 Department of Gynecology and Obstetrics, Heidelberg University Women’s Hospital, INF 440, 69120 Heidelberg, Germany; 2 National Center for Tumor Diseases (NCT), Heidelberg University Hospital, INF 460, 69120 Heidelberg, Germany; 3 Institute of Medical Biometry and Informatics, University of Heidelberg, INF 130.3, 69120 Heidelberg, Germany

**Keywords:** Breast cancer, Neoadjuvant chemotherapy, Neutrophil-to-lymphocyte ratio, Pathologic complete response

## Abstract

**Purpose:**

Breast cancer (BC) is the most common malignancy among women and prognosis is strongly influenced by tumor subtype. Neoadjuvant chemotherapy (NAC) is the standard treatment for both locally advanced- and early-stage triple-negative and Her2-positive BC. Pathologic complete response (pCR) to NAC is an important predictor of patient outcomes. Neutrophil-to-lymphocyte-ratio (NLR) in peripheral blood is associated with prognosis in various malignancies. Here, we investigated the value of the pretreatment NLR as a response predictor in neoadjuvant-treated patients with BC.

**Methods:**

A retrospective chart analysis of 862 patients with invasive BC treated with NAC at the Heidelberg University Hospital during 2003–2015 was conducted. NLR was calculated as the ratio of the absolute neutrophil and lymphocyte counts in peripheral blood, and pCR was defined as absence of invasive or in situ carcinoma in breast and axillary lymph nodes.

**Results:**

A total of 151 patients with invasive BC who underwent NAC were included in this study. NLR tended to be higher in the pCR group than the non-pCR group (*p* < 0.1). Analyses of BC subtypes demonstrated that NLR was significantly higher in the pCR- compared with the non-pCR group (3.304 vs. 2.379, respectively; *p* = 0.048) in patients with luminal B/Her2-negative tumors. Further, we found a significant difference in NLR according to remission status in postmenopausal patients (2.861 vs. 2.313, respectively; *p* = 0.043).

**Conclusion:**

NLR was significantly higher only for patients achieving pCR in the Luminal B/Her2-negative and postmenopausal subgroups. Hence, NLR is a candidate additional predictive factor in patients with Luminal B/Her2-negative BC.

## What does this study add to the clinical work

**Table:** 

A high NLR in patients with early breast cancer may correlate with a good response to neoadjuvant chemotherapy. In our study, we observed this effect in postmenopausal patients and patients with Luminal B/Her2-negative tumors.	

## Introduction

Breast cancer (BC) is the most common malignancy among women worldwide [1]. BC intrinsic subtype (Luminal A, Luminal B/Her2-positive, Her2-negative, Her2-enriched, and Triple-negative subtype), tumor characteristics (e.g., tumor size, nodal status), patient characteristics (e.g., age, menopausal status), and particularly response to treatment are important parameters in estimating patient prognosis [2, 3]. Regarding response to treatment, pathologic complete response (pCR) to neoadjuvant chemotherapy is of particular prognostic value for determining the outcomes of patients with BC. Recent trials showed that patients with several BC subtypes who attain pCR have improved survival; however, not all patients benefited equally [4–7].

According to current research, the host immune system also plays a crucial role in cancer development, progression, and metastasis [8, 9]. Systemic inflammatory markers, such as platelet-to-lymphocyte-ratio, neutrophil-to-lymphocyte-ratio (NLR), and lymphocyte-to-monocyte-ratio, have been reported to correlate with prognosis or pCR in various types of malignancy, including BC [10–14]. Some years ago, Templeton and colleagues assessed the prognostic effect of NLR undertaking a meta-analysis of 100 studies comprising 40,559 patients with unselected solid tumors. They found that an elevated NLR was associated with decreased overall survival (hazard ratio 1.81; 95% confidence interval = 1.67–1.97; *p* < 0.001) with the highest NLR in mesothelioma (hazard ratio 2.35; 95% confidence interval = 1.89–2.92), followed by pancreatic cancer (hazard ratio = 2.27; 95% confidence interval = 1.01–5.14) and renal cell carcinoma (hazard ratio = 2.22; 95% confidence interval = 1.01–5.14). [15]

While lymphocytes are important key contributors to immune reactions against tumors, neutrophils can suppress the anti-tumor activity of lymphocytes and promote tumor cell migration, as well as angiogenesis [16–18]. These blood-based parameters could provide an additional, cost-effective, and easy to perform method of risk assessment.

The main aim of this study was to investigate the role of the NLR as a response predictor in neoadjuvant-treated patients with BC.

## Patients and methods

### Study design and patients

**Fig. 1:**
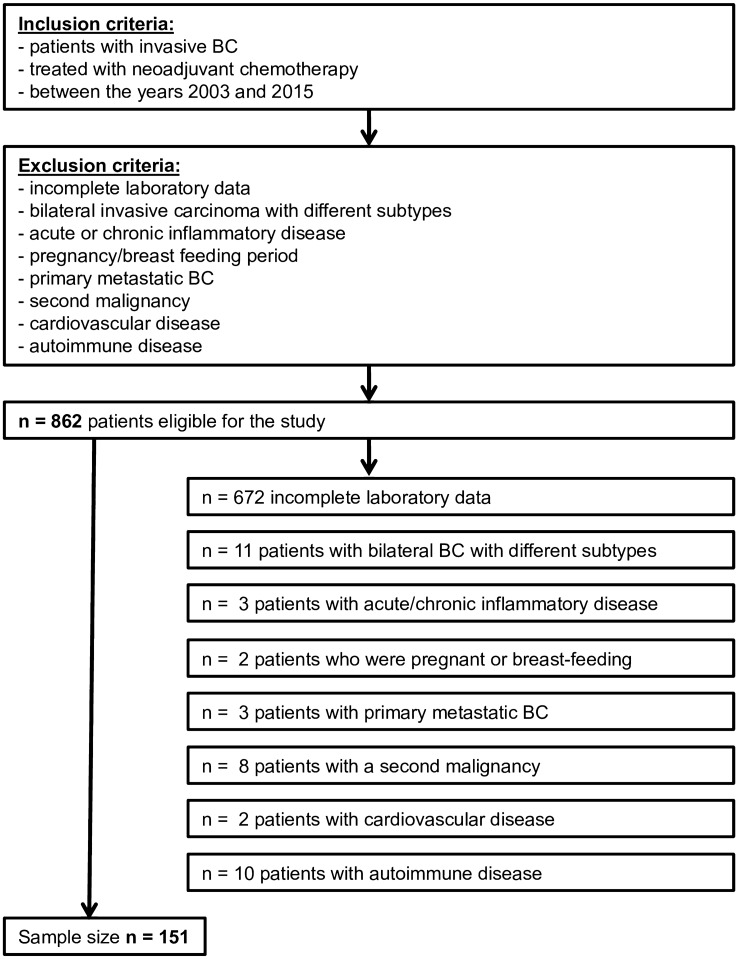
Flow chart: Study design and patient enrollment process; eligibility criteria

In this study, we performed a retrospective chart analysis of 862 patients with invasive BC who were treated with neoadjuvant chemotherapy at the Heidelberg University Hospital in the years 2003–2015. Data were collected using the patient data management program ISHmed^®^ (SAP GmbH Walldorf, Germany). As shown in Fig. 1, according to the exclusion criteria, 711 patients were excluded: 672 because of incomplete laboratory data, 11 with bilateral invasive BC with various cancer phenotypes, 10 with autoimmune disease, 8 with a secondary malignancy, 3 with acute or chronic inflammatory disease, 2 with cardiovascular disease, 3 due to primary metastasis, and 2 because they were pregnant or breast feeding. Finally, the medical records of 151 patients with BC were eligible for inclusion in the present investigation.

Concerning patient characteristics, we collected data on patient medical history, age, body mass index (BMI), menopausal status, tumor characteristics (size, stage, nodal status, histopathological characteristics), and laboratory results (absolute neutrophil count, absolute monocyte count, absolute platelet count, absolute lymphocyte count). Using a differential blood count performed after BC diagnosis and before treatment initiation, NLR was calculated as the ratio of the absolute neutrophil count to the absolute lymphocyte count.

Estrogen receptor (ER), progesterone receptor (PR), and human epidermal growth factor receptor 2 (Her2) expression were primarily assessed by immunohistochemistry. Fluorescent or chromogenic in situ hybridization was performed for cases with intermediate Her2 scores (2 +). According to the definition of Goldhirsch et al., we used clinicopathological parameters to define the BC subtypes: luminal A (ER + , PR ± , Her2-, Ki-67 < 20%), luminal B/Her2-negative (ER + , PR ± , Her2-, Ki-67 ≥ 20%), luminal B/Her2-positive (ER + , PR ± , Her2 +), Her2-enriched (ER-, PR-, Her2 +), and triple-negative BC (TNBC) (ER-, PR-, Her2-) [18]. A pCR was defined as the total absence of invasive or in situ carcinoma within the breast and axillary lymph nodes (ypT0 ypN0).

The present investigation was approved by the University of Heidelberg Ethics Committee (S-094/2017).

### Statistics

Accrued data were pseudonymized and analyzed using SPSS statistics version 24.0 (SPSS Inc., Chicago, USA). Data are presented as mean ± standard deviation (SD) (unless stated otherwise) in case of continuous variables and as absolute and relative frequencies in case of categorical variables. For statistical comparisons of mean values, t tests or ANOVA models were applied. To assess differences in ordinal variables Mann–Whitney *U *tests were used, Pearson’s chi-squared tests were applied in case of nominal (categorical) variables. The level of significance was set to 5%; therefore, a *p* value of < 0.05 was considered statistically significant; for *p* values of < 0.1, a tendency toward difference was assumed. Since this was an exploratory study, we did not apply any adjustment for multiplicity.

## Results

### Patient and tumor characteristics

From 2003 to 2015, a total of 862 patients with invasive BC who underwent neoadjuvant chemotherapy were eligible for this study. Based on the exclusion criteria, 711 patients were not included in the statistical analyses (Fig. 1). Hence, the total study cohort comprised 151 patients. As shown in Table 1, the mean age at primary diagnosis was 50 years (± 11.03; range 27–77 years). The majority (58.9%, *n* = 89) of our cohort were premenopausal, while 41.1% (*n* = 62) were postmenopausal. At 55% (*n* = 83), the majority of our patients had an initial tumor size of cT2, while 23.2% (*n* = 35), 11.2% (*n* = 17), and 10.6% (*n* = 16) had cT1-, cT3-, and cT4-stage tumors, respectively (Table 1). At primary diagnosis, 87 patients were node-negative (cN0; 57.6%). Regarding the tumor subtypes in our cohort, 42 patients (27.8%) had luminal B/Her2-negative, 36 (23.8%) luminal B/Her2-positive, 56 (37.1%) triple-negative, and 17 (11.3%) Her2-enriched subtype tumors. There were no luminal A tumors in our cohort. All patients were treated with standardized neoadjuvant chemotherapy, which in most cases consisted of taxane + anthracycline and/or cyclophosphamide (Table 1). All Her2-positive patients were additionally treated with trastuzumab. A total of 66 patients achieved pCR (43.7%) while 85 (56.3%) showed residual invasive or in situ carcinoma. The highest pCR rate (88%; *n* = 15/17) was observed for patients with Her2-enriched subtype tumors.

**Table 1:** Characteristics of patients according to pathological complete response (pCR) versus non-pCR and total cohort

Characteristic	Total cohort (*n* = 151)	pCR (*n* = 66)	Non-pCR (*n* = 85)	*p* value
Age (years)				0.027*^1^
Mean ± SD	49.97 ± 11.03	48.08 ± 11.0	52.06 ± 10.7	
Median	49	47.5	50	
Minimum	27	27	29	
Maximum	77	74	77	
BMI (kg/m^2^)				0.624*^1^
Mean ± SD	25.7 ± 4.56	25.5 ± 4.51	25.9 ± 4.62	
Median	25.1	25.3	24.7	
Minimum	18.7	18.7	18.7	
Maximum	41.5	41.5	39.0	
Menopausal status				0.042*^2^
Premenopausal	89 (58.9%)	45 (68.2%)	44 (51.8%)	
Postmenopausal	62 (41.1%)	21 (31.8%)	41 (48.2%)	
Smoking behavior				0.531*^2^
Smoker	33 (21.9%)	16 (24.2%)	17 (20%)	
Non-smoker	118 (78.1%)	50 (75.8%)	68 (80%)	
cT-stage				0.013*^3^
T1	35 (23.2%)	22 (33.3%)	13 (15.3%)	
T2	83 (55.0%)	34 (51.5%)	49 (57.7%)	
T3	17 (11.2%)	5 (7.6%)	12 (14.1%)	
T4	16 (10.6%)	5 (7.6%)	11 (12.9%)	
cN-status				0.099*^2^
Positive cN +	64 (42.4%)	23 (34.8%)	41 (48.2%)	
Negative cN0	87 (57.6%)	43 (65.2%)	44 (51.8%)	
Grading				0.009*^2^
G2	68 (45.0%)	22 (33.3%)	46 (54.1%)	
G3	82 (54.3%)	44 (66.7%)	38 (44.7%)	
Missing	1 (0.7%)	-	1 (1.2%)	
Histology				0.004*^2^
Ductal carcinoma	141 (93.4%)	66 (100%)	75 (88.2%)	
Others *^4^	10 (6.6%)	0 (0%)	10 (11.8%)	
Estrogen receptor status				0.001*^2^
Negative	78 (51.7%)	44 (66,7%)	34 (40%)	
Positive	73 (48.3%)	22 (33,3%)	51 (60%)	
Progesterone receptor				0.004*^2^
Negative	83 (55.0%)	45 (68.2%)	38 (44.7%)	
Positive	68 (45.0%)	21 (31.8%)	47 (55.3%)	
Her2 receptor status				0.002*^2^
Negative	98 (64.9%)	34 (51.5%)	64 (75.3%)	
Positive	53 (35.1%)	32 (48.5%)	21 (24.7%)	
Intrinsic phenotype				0.005*^3^
Lum B/Her2-negative	42 (27.8%)	8 (12.1%)	34 (40.0%)	
Lum B/Her2-positive	36 (23.8%)	17 (25.8%)	19 (22.35%)	
Her2-enriched	17 (11.3%)	15 (22.7%)	2 (2.35%)	
Triple-negative	56 (37.1%)	26 (39.4%)	30 (35.3%)	
Neoadjuvant chemotherapy				0.160*^3^
Taxane/anthracycline/cyclophosphamide	101 (66.9%)	46 (69.7%)	55 (64.7%)	
Taxane/anthracycline	31 (20.5%)	14 (21.2%)	17 (20.0%)	
Taxane/cyclophosphamide	9 (6.0%)	5 (7.6%)	4 (4.7%)	
Other regimen	10 (6.6%)	1 (1.5%)	9 (10.6%)	

The percentage rates correspond to the different patient groups (total cohort, pCR group, and non-pCR group). Significant values are indicated in bold. *n* = number

*^1^Student’s *t* test (independent samples *t* test). *^2^Chi-squared test. *^3^Mann–Whitney U test. *^4^Invasive lobular carcinoma, inflammatory BC, neuroendocrine BC

### NLR

We investigated associations of NLR with both clinical and histopathological parameters and found that it was significantly higher in younger patients (age < 50 years, NLR = 3.129 ± 1.725 vs. age ≥ 50 years, 2.628 ± 1.225; *p* = 0.042), premenopausal patients (premenopausal patients, mean NLR = 3.151 ± 1.742 vs. postmenopausal patients 2.499 ± 1.014; *p* = 0.009), and patients with BMI < 25 kg/m^2^ (BMI < 25 kg/m^2^, NLR = 3.186 ± SD 1.793 vs. BMI ≥ 25 kg/m^2^ 2.655 ± 1.232; *p* = 0.033). Furthermore, patients with Her2-positive tumors also had significantly higher NLR values than those with Her2-negative tumors (3.263 ± 1.546 vs. Her2-negative 2.678 ± 1.468; *p* = 0.023). No significant differences in NLR were detected according to ER- and PR-status (Table 2).

**Table 2:** Associations of NLR with clinical and histopathological parameters in the total cohort (*n* = 151)

Patient characteristic	Total cohort (*n* = 151)	
	NLR (mean)	*p* value
Age		0.042*^1^
< 50 years (*n* = 77)	3.219	
≥ 50 years (*n* = 74)	2.628	
BMI		0.033*^1^
< 25 kg/m^2^ (*n* = 65)	3.186	
≥ 25 kg/m^2^ (*n* = 86)	2.655	
Menopausal status		0.009*^1^
Premenopausal (*n* = 89)	3.151	
Postmenopausal (*n* = 62)	2.499	
Smoking behavior		0.735*^1^
Smoker (*n* = 33)	2.963	
Non-smoker (*n* = 118)	2.861	
cT-status		0.178*^1^
cT1/2 (*n* = 117)	2.794	
cT3/4 (*n* = 34)	3.192	
cN-status		0.474*^1^
cN0 (*n* = 87)	2.807	
cN + (*n* = 64)	2.987	
ypT-stage		0.154*^1^
ypT0 (*n* = 74)	3.063	
ypT1/2/3/4 (*n* = 77)	2.710	
ypN-status		0.244*^1^
ypN0 (*n* = 121)	2.819	
ypN1/2/3 (*n* = 29)	3.187	
Missing (*n* = 1)		
Grading		0.948*^1^
G2 (*n* = 68)	2.881	
G3 (*n* = 82)	2.898	
Missing * n* = 1		
Histology		0.888*^1^
Ductal carcinoma (*n* = 141)	2.888	
Others (*n* = 10)	2.818	
Ki-67		0.409*^1^
≤ 20% (*n* = 20)	3.145	
> 20% (*n* = 131)	2.843	
Her2 receptor status		0.023*^1^
Negative (*n* = 98)	2.678	
Positive (*n* = 53)	3.263	
Estrogen receptor status		0.865*^1^
Positive (*n* = 73)	2.905	
Negative (*n* = 78)	2.863	
Progesterone receptor status		0.767*^1^
Positive (*n* = 68)	2.924	
Negative (*n* = 83)	2.850	
Intrinsic phenotype		0.121*^2^
Luminal B/Her2-negative (*n* = 42)	2.555	
Luminal B/Her2-positive (*n* = 36)	3.327	
Her2-enriched (*n* = 17)	3.126	
Triple-negative (*n* = 56)	2.771	
Remission status		0.095*^1^
pCR (*n* = 66)	3.118	
Non-pCR (*n* = 85)	2.702	

Significant values are indicated in bold. *n* = number; *^1^Student’s t test (independent samples t test). *^2^ Univariate ANOVA

Our primary objective was to investigate the association between NLR and pCR. Hence, we divided our cohort into two subgroups: pCR and non-pCR. With a mean ratio of 3.118 ± 1.783, NLR tended to be higher in the pCR group than the non-pCR-group (NLR = 2.702 ± 1.253); however, the difference was not statistically significant (*p* = 0.095) (Table 2). To better understand the role of BC subtype, we conducted a sub-analysis. The results demonstrated that, for luminal B/Her2-negative patients, NLR was also higher in the pCR-subgroup compared with the non-pCR-subgroup; however, the difference was significant in this case (3.304 ± 1.582 vs. non-pCR 2.378 ± 1.0435; *p* = 0.048). Regarding the TNBC, luminal B/Her2-positive, and Her2-enriched subtypes, no association was detected between NLR and achieving pCR.

Since we found differences in NLR depending on age and menopausal status, we performed sub-analyses for the premenopausal (*n* = 89) and postmenopausal (*n* = 62) patient cohorts (Table 3). In the postmenopausal cohort, NLR was significantly higher among patients achieving pCR (2.861 ± 1.068) than for those who did not (non-pCR) (2.313 ± 0.945; *p* = 0.043). Furthermore, we also found that NLR was significantly higher among postmenopausal patients with luminal B/Her2-negative subtype tumors who achieved pCR (5.338 ± 0.370) compared with those who did not (2.362 ± 1.241; *p* = 0.004). For the premenopausal cohort, we did not detect any significant differences in NLR among the different subsets (Table 3).

**Table 3:** Association of NLR with clinical and histopathological parameters in the postmenopausal and premenopausal cohorts

Characteristic (*n* premenopausal/*n* postmenopausal)	Premenopausal (*n* = 89)		Postmenopausal (*n* = 62)	
	NLR (mean)	*p* value	NLR (mean)	*p* value
Age		0.758*^1^		0.585*^1^
< 50 years (*n* = 73/*n* = 4)	3.178		2.228	
≥ 50 years (*n* = 16/*n* = 58)	3.029		2.517	
BMI		0.205*^1^		0.332*^1^
< 25 kg/m^2^ (*n* = 47/*n* = 18)	3.373		2.695	
≥ 25 kg/m^2^ (*n* = 42/*n* = 44)	2.903		2.418	
Smoking behavior		0.630*^1^		0.151*^1^
Smoker (*n* = 22/*n* = 11)	3.203		2.898	
Non-smoker (*n* = 67/*n* = 51)	2.995		2.412	
cT-status		0.066*^1^		0.670*^1^
cT1/2 (*n* = 73/*n* = 44)	2.993		2.463	
cT3/4 (*n* = 16/*n* = 18)	3.875		2.585	
cN-status		0.864*^1^		0.152*^1^
cN0 (*n* = 52/*n* = 35)	3.125		2.336	
cN + (*n* = 37/*n* = 27)	3.189		2.710	
ypT-status		0.831*^1^		0.057*^1^
ypT0 (*n* = 50/*n* = 24)	3.187		2.807	
ypT1/2/3/4 (*n* = 39/*n* = 38)	3.106		2.304	
ypN-status		0.483*^1^		0.190*^1^
ypN0 (*n* = 72/*n* = 49)	3.088		2.424	
ypN1/2/3 (*n* = 17/*n* = 12)	3.420		2.856	
Missing (*n* = 1)				
Grading		0.732*^1^		0.424*^1^
G2 (*n* = 40/*n* = 28)	3.222		2.395	
G3 (*n* = 49/*n* = 33) Missing (*n* = 1)	3.094		2.607	
Histology		0.499*^1^		0.469*^1^
Ductal carcinoma (*n* = 84/*n* = 57)	3.173		2.468	
Others (*n* = 1/*n* = 4)	1.955		2.855	
Missing (*n* = 4/*n* = 1)				
Ki-67		0.111*^1^		0.946*^1^
≤ 20 (*n* = 8/*n* = 12)	4.088		2.517	
> 20 (*n* = 81/*n* = 50)	3.059		2.494	
Her2 receptor status		0.060*^1^		0.372*^1^
Positive (*n* = 34/*n* = 19)	3.591		2.673	
Negative (*n* = 55/*n* = 43)	2.879		2.422	
Estrogen receptor status		0.758*^1^		0.384*^1^
Positive (*n* = 44/*n* = 29)	3.093		2.619	
Negative (*n* = 45/*n* = 33)	3.208		2.393	
Progesterone receptor status		0.953*^1^		0.411*^1^
Positive (*n* = 40/*n* = 28)	3.139		2.616	
Negative (*n* = 49/*n* = 34)	3.161		2.402	
Intrinsic phenotype		0.109*^2^		0.414*^2^
Luminal B/Her2-negative (*n* = 22/*n* = 20)	2.459		2.660	
Luminal B/Her2-positive (*n* = 22/*n* = 14)	3.727		2.700	
Her2-enriched (*n* = 12/*n* = 5)	3.344		2.603	
Triple-negative (*n* = 33/*n* = 23)	3.159		2.215	
Luminal B/Her2-negative (*n* = 22/*n* = 20)		0.600*^1^		0.004*^1^
pCR (*n* = 6/*n* = 2)	2.626		5.338	
Non-pCR (*n* = 16/*n* = 18)	2.397		2.362	
Luminal B/Her2-positive (*n* = 22/*n* = 14)		0.989*^1^		0.654*^1^
pCR (*n* = 9/*n* = 8)	3.720		2.626	
Non-pCR (*n* = 13/*n* = 6)	3.733		2.794	
Her2-enriched (*n* = 12/*n* = 5)		0.634*^1^		– *^3^
pCR (*n* = 10/*n* = 0)*^3^	3.441		–	
Non-pCR (*n* = 2/*n* = 5)	2.858		2.603	
Triple-negative (*n* = 33/*n* = 23)		0.843*^1^		0.150*^1^
pCR (*n* = 20/*n* = 6)	3.102		2.561	
Non-pCR (*n* = 13/*n* = 17)	3.246		2.092	
Remission status		0.640*^1^		0.043*^1^
pCR (*n* = 45/*n* = 21)	3.237		2.861	
Non-pCR (*n* = 44/*n* = 41)	3.063		2.313	

Significant values are indicated in bold. *n* = number; *^1^Student’s *t* test (independent samples t test). *^2^Univariate ANOVA. *^3^No testing possible

## Discussion

The present investigation focused on the role of NLR as a response predictor in neoadjuvant-treated patients with different BC subtypes. Several previous reports have suggested that patients with cancer who have an elevated pretreatment NLR have worse prognosis than those with low values for this parameter [10–12]; however, the data relating to NLR in BC are heterogeneous [20]. On the one hand, Noh and Han found that there was a significant correlation between a low NLR and superior disease-specific survival in patients with BC, although analysis by subtype only detected a significant difference for the luminal A cohort [12]. On the other hand, Patel et al. showed that low NLR was significantly associated with longer overall survival in TNBC patients. [21] In contrast, Suppan et al. and Goto et al. did not detect an association between NLR and disease-free survival, independent of the BC subtype [22, 23].

In our study, we found that elevated pretreatment NLR tended to be associated with a pCR after neoadjuvant chemotherapy, which was significant for postmenopausal patients and those with luminal B/Her2-negative tumors. As described above, pCR after neoadjuvant chemotherapy seems to be of prognostic value for determining better outcomes of patients with BC [4, 6]. Initially, this may appear to be contradictive to the trials that found that a high NLR was associated with a worse prognosis. However, as patients with more aggressive tumors are more likely to achieve a pCR, this may not be the case. Characteristics, such as a high proliferation index, high tumor grade, triple-negative or Her2-enriched subtype or young age at primary diagnosis, are associated with higher rates of pCR [4, 24]. In our patient cohort, we also found that the tumor grade G3, younger age (< 50 years), premenopausal status, and Her2-enriched phenotype were significantly associated with high NLR; these findings are consistent with the current literature [12, 23]. Those characteristics are routinely used as part of the process of indication for undergoing neoadjuvant chemotherapy. While patients with triple-negative or Her2-positive tumors are currently routinely treated with (neo-) adjuvant chemotherapy (± Her2-targeted antibody therapy), the optimal treatment for the luminal B/Her2-negative subgroup of patients remains under discussion. In clinical practice, the original molecularly defined BC subtypes are routinely classified by a pathological examination, according to Goldhirsch et al. [25]; however, using immunohistochemical methods, clear differentiation between luminal A-like and luminal B-like tumors can be challenging. Recently, several gene expression tests, such as Prosigna or Oncotype DX, have been developed as an additional aid to treatment decisions concerning adjuvant chemotherapy in patients with early luminal BC [26]. Although the treatment recommendations are relatively clear for patients with low or high risk of recurrence scores, the interpretation of intermediate risk of recurrence scores remains under investigation. Moreover, to date, gene expression tests are performed using breast tumor specimens usually collected intraoperatively, which stands against the possibility of neoadjuvant treatment. Consequently, those patients may not benefit from the advantages of a neoadjuvant therapy, such as down-staging of tumors with consecutively reduced surgical invasiveness or in vivo analysis of sensitivity to treatment [27]. Combined with the fact that pCR is a suitable predictor of superior outcome, the results of our study, demonstrating that patients with luminal B/Her2-negative tumors achieving pCR had a significantly higher pretreatment NLR, it is possible that patients with elevated NLR, and particularly those with luminal B/Her2-negative tumors, benefit from neoadjuvant treatment.

Still our study has several limitations. Specifically, the investigation was retrospective in design and the sample size was very small; although we investigated a total of 862 patients, 672 were excluded because of insufficient laboratory data. Therefore, data interpretation is only possible to a limited extent and the results should first be verified on a larger cohort. Furthermore, we do not have survival outcome data for the majority of eligible patients. Hence, we could not compare the actual survival data with the prognostic value of pCR.

In conclusion, our results suggest that, for postmenopausal patients with BC and those with luminal B/Her2-negative subtype tumors, pretreatment NLR may provide additional information regarding the likelihood of achieving a pathologic complete response to neoadjuvant chemotherapy. As pCR is a suitable surrogate end point, we assume that pretreatment NLR could be considered as an additional predictive factor for patients with luminal B/Her2-negative subtype BC. Future prospective studies are necessary to confirm our findings in a larger patient cohort and further evaluate the underlying molecular mechanism.
